# WNT/β-Catenin-Mediated Resistance to Glucose Deprivation in Glioblastoma Stem-like Cells

**DOI:** 10.3390/cancers14133165

**Published:** 2022-06-28

**Authors:** Suad Yusuf, Philippe Aretz, Ann-Christin Nickel, Philipp Westhoff, Amit Sharma, Nan Qin, Marc Remke, Hans-Jakob Steiger, Daniel Hänggi, Hongjia Liu, Hongde Liu, Silke Neumann, Guido Reifenberger, Jarek Maciaczyk

**Affiliations:** 1 Department of Neurosurgery, Medical Faculty, University Hospital Düsseldorf, Heinrich Heine University, 40225 Düsseldorf, Germany; suad.yusuf@uni-duesseldorf.de (S.Y.); philippe.aretz@uni-duesseldorf.de (P.A.); ann-christin.nickel@uni-duesseldorf.de (A.-C.N.); daniel.haenggi@med.uni-duesseldorf.de (D.H.); 2 Plant Metabolism and Metabolomics Laboratory, Heinrich Heine University, 40225 Düsseldorf, Germany; philipp.westhoff@hhu.de; 3 Department of Stereotactic and Functional Neurosurgery, Medical Faculty, University Hospital Bonn, University of Bonn, 53127 Bonn, Germany; amit.sharma@ukbonn.de; 4 Department of Pediatrics, Medical Faculty, University Hospital Düsseldorf, Heinrich Heine University, 40225 Düsseldorf, Germany; nan.qin@med.uni-duesseldorf.de (N.Q.); marc.remke@med.uni-duesseldorf.de (M.R.); 5 Department of Neurosurgery, University of Basel, Canton Hospital Aarau, 5001 Aarau, Switzerland; hsteiger@uni-duesseldorf.de; 6 State Key Laboratory of Bioelectronics, School of Biological Science & Medical Engineering, Southeast University, Nanjing 210096, China; liuhongjia@seu.edu.cn (H.L.); liuhongde@seu.edu.cn (H.L.); 7 Department of Pathology, Dunedin School of Medicine, University of Otago, Dunedin 9016, New Zealand; silke.neumann@otago.ac.nz; 8 Institute of Neuropathology, Medical Faculty, University Hospital Düsseldorf, Heinrich Heine University, 40225 Düsseldorf, Germany; reifenberger@med.uni-duesseldorf.de; 9 Department of Surgical Sciences, Dunedin School of Medicine, University of Otago, Dunedin 9016, New Zealand

**Keywords:** glioblastoma, cancer stem-like cells, glucose starvation, cancer metabolism, WNT/β-catenin

## Abstract

**Simple Summary:**

The malignant growth and therapy resistance of isocitrate dehydrogenase (IDH)-wildtype glioblastoma is thought to be driven by a subpopulation of tumor cells with cancer stem-like cell (CSC) properties. Employing a high-throughput in vitro drug screen, we identified LGK974 and berberine as drugs that impair wingless (WNT) signaling and can thereby sensitize glioblastoma stem-like cells (GSCs) to glucose starvation-induced cell death. The main goal of this study was to characterize the role of the WNT pathway in mediating the survival and metabolic plasticity of GSCs under nutrient-restricted growth conditions. Gas chromatography mass spectrometry (GC-MS) was used to determine WNT-specific alterations of intracellular metabolites in GSCs grown under nutrient restriction, i.e., glucose depletion, or under standard conditions. Metabolic fingerprints hold the promise to complement classic biomarkers, thus potentially aiding the prediction of tumor behavior and patient prognosis.

**Abstract:**

Isocitrate dehydrogenase (IDH)-wildtype glioblastoma is the most common primary malignant brain tumor. It is associated with a particularly poor prognosis, as reflected by an overall median survival of only 15 months in patients who undergo a supramarginal surgical reduction of the tumor mass followed by combined chemoradiotherapy. The highly malignant nature of IDH-wildtype glioblastoma is thought to be driven by glioblastoma stem-like cells (GSCs) that harbor the ability of self-renewal, survival, and adaptability to challenging environmental conditions. The wingless (WNT) signaling pathway is a phylogenetically highly conserved stemness pathway, which promotes metabolic plasticity and adaptation to a nutrient-limited tumor microenvironment. To unravel the reciprocal regulation of the WNT pathway and the nutrient-limited microenvironment, glioblastoma cancer stem-like cells were cultured in a medium with either standard or reduced glucose concentrations for various time points (24, 48, and 72 h). Glucose depletion reduced cell viability and facilitated the survival of a small population of starvation-resistant tumor cells. The surviving cells demonstrated increased clonogenic and invasive properties as well as enhanced chemosensitivity to pharmacological inhibitors of the WNT pathway (LGK974, berberine). Glucose depletion partially led to the upregulation of WNT target genes such as *CTNNB1*, *ZEB1*, and *AXIN2* at the mRNA and corresponding protein levels. LGK974 treatment alone or in combination with glucose depletion also altered the metabolite concentration in intracellular compartments, suggesting WNT-mediated metabolic regulation. Taken together, our findings suggest that WNT-mediated metabolic plasticity modulates the survival of GSCs under nutrient-restricted environmental conditions.

## 1. Introduction

Gliomas are the most common primary malignant brain tumors [1]. They are classified into distinct tumor types based on the combination of histopathological features and molecular biomarkers according to the World Health Organization (WHO) classification of central nervous system (CNS) tumors [2]. Among the diffusely infiltrating gliomas in adults, the WHO classification distinguishes three major tumor types, namely the IDH-mutant astrocytomas of CNS WHO grades 2, 3, or 4, the IDH-mutant and 1p/19q-codeleted oligodendrogliomas of CNS WHO grades 2 or 3, as well as the IDH-wildtype glioblastomas of CNS WHO grade 4. Malignant glioma growth and resistance to therapy are thought to be driven by a subpopulation of tumor cells with stem-like features, including the ability of self-renewal and an adaptability to extreme and adverse microenvironmental conditions in terms of nutrient availability, especially during rapid tumor growth [3,4,5,6]. Tumor expansion leads to hypovascularized, hypoxic, and nutrient-deprived tumor areas consequently favoring the survival of subpopulations of nutrient stress-resilient cells [7]. The study of the metabolic plasticity and reprogramming of tumor cells in nutrient-limited microenvironments in IDH-wildtype glioblastoma is a promising area of research that may delineate mechanisms promoting tumor cell survival [8]. Stress resilience has been attributed to stem-like tumor cells in glioblastomas, with stemness playing an essential role in promoting the resilience, self-renewal, and metabolic adaptability of glioblastoma cells [9]. Various stemness factors and phylogenetically conserved stemness pathways, such as the WNT pathway, have been implicated as drivers of cancer cell stemness and tumor growth in different types of cancers, especially breast cancer, colorectal cancer, prostate cancer, and glioblastoma [10]. WNT signaling has pleiotropic effects on cell physiology and development during embryogenesis and organogenesis, especially in nervous tissue, and in maintaining metabolic homeostasis. WNT signaling can be divided into several independent pathways such as the WNT/β-catenin-dependent (referred to as the canonical WNT pathway) and the non-canonical pathway. The non-canonical pathway is further subdivided into the planar cell polarity and the WNT/Ca^2+^ pathway, each inducing different downstream cascades and thereby promoting different cellular effects. The most studied WNT pathway is the canonical WNT signaling pathway, which regulates the abundancy of the transcriptional co-activator β-catenin that controls key developmental processes [11]. Altered β-catenin signaling, especially via the aberrant activation of the canonical WNT pathway, plays an important role in promoting tumor growth in various tumor types, including glioblastoma [12]. Recently, our group has shown that β-catenin, together with CCL2, promotes monocyte migration towards glioblastoma cells [13]. In particular, WNT-mediated β-catenin signaling is thought to promote metabolic changes in cancer [14]. For example, the transcriptional upregulation of monocarboxylate-transporter 1 (MCT-1) has been linked to β-catenin-mediated promoter activation via binding to *TCF*/*LEF* sites, which consequently leads to lactate secretion and increased aerobic glycolysis [15]. It has been discussed that activation of the canonical WNT pathway may lead to the activation of aerobic glycolysis through the upregulation of pyruvate dehydrogenase (PDH), pyruvate kinase M2 (PKM2), lactate dehydrogenase A (LDH-A), a sodium-dependent unspecific amino acids transporter (SLC1A5), and glucose transporter 1 (GLUT1). Non-canonical WNT signaling also enhances glycolysis by activating the Akt-mTOR pathway [14]. Fatty acid oxidative metabolism is also enhanced by mTORC1 and β-catenin signaling [16]. In addition, glucose-6-phosphate dehydrogenase, increased by mTOR as a consequence of an upregulated pentose phosphate bypass, leads to the enhanced availability of reduction-equivalents, such as co-enzyme nicotinamide adenine dinucleotide phosphate (NADPH/H^+^), and provides phosphoribosyl pyrophosphate (PRPP) for purine and pyrimidine synthesis, thus supplying more substrates for efficient DNA synthesis [17].

In the present study, we focused on cellular models of IDH-wildtype GSCs and investigated the impact of glucose concentration on the cellular metabolism and biological features of GSCs in vitro. As a proof of principle, we assessed the effects of glucose deprivation on the WNT/β-catenin pathway in glioblastoma and evaluated the consequences of pharmacological WNT inhibition on GSCs under glucose-restricted conditions.

## 2. Materials and Methods

### 2.1. Cell Culture and Glucose Starvation

We used three GSCs models in our study: GBM1 cells (generously provided by A. Vescovi, San Raffaele Hospital, Milano, Italy), JHH520 cells (generously provided by G. Riggins, Johns Hopkins, Baltimore, MD, USA), and BTSC233 cells (generously provided by M.S. Carro, Freiburg University, Freiburg im Breisgau, Germany). As a standard protocol for glioblastoma stem-like cancer cell enrichment, we cultured these cell lines in Neurobasal™-A medium without D-glucose and sodium pyruvate, and substituted glucose equivalent to the standard cell culture glucose concentration (450 mg/dL) using a glucose solution (200 g/L; Gibco BRL). The starvation protocol was conducted as follows: 5 mL of cells cultured in standard cell culture glucose concentration (450 mg/dL) were collected and centrifuged for 5 min (min) at 1000 rpm. The supernatant was then removed and the harvested cells were resuspended in Neurobasal^TM^-A complete medium without D-glucose for a defined period of time. In addition, 2% B27 supplement (Gibco), 20 ng/mL human bFGF (Peprotech, Rocky Hill, NJ, USA), 20 ng/mL human EGF (Peprotech), 5 µg/mL heparin (Sigma, Merck KGaA, Darmstadt, Germany), and 1% penicillin–streptomycin–fungicide mixture (Gibco) were added to the medium. The cell lines were cultured at 37 °C and 5% CO_2_.

### 2.2. Cell Viability

To evaluate cell viability, 5 × 10^3^ cells of each glioblastoma cell line were resuspended in 100 µL Neurosphere complete medium and seeded to a 96-well flat-bottom suspension plate. The 100 µL of Neurosphere complete medium was adjusted to different glucose concentrations: 100% (450 mg/dL), 50% (225 mg/dL), 25% (112.5 mg/dL), 10% (45 mg/dL), and 0% (0 mg/dL) of standard cell culture glucose concentration using Gibco glucose solution 200 g/L (Gibco BRL) and cultured for 2, 24, and 48 h. Cell viability was determined using the thiazolyl blue tetrazolium bromide assay (MTT, Sigma-Aldrich). The samples were measured on a Paradigm™ multiplate reader (Beckman Coulter, Brea, CA, USA) at wavelengths of 570 nm and 650 nm.

### 2.3. Invasion Assay

The ability of glucose-starved GSCs to invade through a Matrigel-coated membrane was evaluated using a modified Boyden chamber assay, as previously described in reference [18]. The inserts (Falcon) were coated with Matrigel before cells (1 × 10^5^/500 µL) were resuspended in Neurobasal complete medium either with or without glucose. The inserts were placed in a 24-well plate and coated with 500 µL of growth factor-reduced Matrigel (BD, Franklin Lakes, NJ, USA) diluted in Neurobasal glucose-depleted medium (1:100). The bottom of the 24-well plate was also coated. Following an incubation period of 1 h at 37 °C, the Matrigel was removed, the cells were seeded into the inserts, and the chambers were filled with 800 µL Neurobasal complete medium containing glucose at a standard concentration (450 mg/dL) and 10% FBS. After 48 h, the cell suspensions inside the inserts were removed and the non-invaded cells on the inner membrane of the inserts were cautiously stripped off with a cotton swap drenched in PBS. Next, cells that invaded through the membrane were fixed by adding ice-cold methanol for 10 min. The inserts were washed twice with PBS and cells were stained with hematoxylin for 5 min. Images were taken on a Zeiss microscope (objective: Zeiss Plan S 1.0 × FWD 81 mm), and the number of cells invaded was evaluated with ImageJ 1.8.0. (Rasband, W.S., U.S. National Institutes of Health, Bethesda, MD, USA).

### 2.4. Soft Agar Colony Formation Assay

The clonogenic capacity of GSCs was determined as previously described in reference [17]. Briefly, six-well plates were coated with 1.5 mL of 1% agarose (Life Technologies) in Neurobasal medium (as a bottom layer) and incubated at 37 °C for 1 h. Then, the middle layer containing 0.6% agarose, 5 × 10^3^ cells/well in Neurobasal complete medium (with and without 10% standard glucose concentration) was added. After solidification at room temperature (RT) for 1 h, the top layer was then prepared and incubated at 37 °C and 5% CO_2_ for 4 weeks. Subsequently, 1 mg/mL 4-nitro-tetrazolium chloride (NBT) solution (Sigma-Aldrich) in PBS was added overnight (37 °C) to stain the colonies. ImageJ 1.8.0. (Rasband, W.S., U.S. National Institutes of Health, Bethesda, MD, USA) was used to count the colonies.

### 2.5. Luciferase Reporter Assay

The luciferase reporter assay was performed as previously described in reference [19]. After transfection of GSCs with a stable lentiviral reporter construct comprising seven *TCF* binding sites trailed by a luciferase cassette, puromycin selection (2 µg/mL) was performed. Subsequently, transfected cells were cultured under standard glucose concentrations and glucose deprivation (48 h). The emitted luminescence was measured at a wavelength of 490 nm on a Paradigm™ multiplate reader (Beckman Coulter, Brea, CA, USA) and normalized to β-galactosidase activity.

### 2.6. Western Blot

Cells were washed with PBS and lysed in ice-cold RIPA buffer. Protein concentrations were quantified using the DC Protein Assay Kit (BioRad, Hercules, CA, USA) and readout was performed using the Paradigm™ Multiplate Reader (Beckman Coulter, Brea, CA, USA) at a wavelength of 750 nm. The subsequent steps were performed as previously described in reference [20]. Primary antibodies: active β-catenin 1:1000 (non-phospho/active β-catenin, Ser33/37/Thr41, rabbit mAb, Cell Signaling, Danvers, MA, USA) and GAPDH 1:5000 (GAPDH, D4C6R, mouse mAb; Cell Signaling, Danvers, MA) were diluted in 5% BSA and incubated overnight at 4 °C on the membranes. Secondary antibodies: goat anti-rabbit IRDye800CW (1:10,000, LI-COR #926-32211) and goat anti-mouse IRDye680RD (1:10,000, LI-COR #926-68070) were diluted in 5% BSA and incubated for 1 h at RT. The fluorescence was assessed using LI-COR Odyssey CLx imager followed by densitometry. GAPDH was used as a housekeeping protein for normalization. Original western blot images can be found in the Supplementary Materials.

### 2.7. Immunostaining for Active β-Catenin

Immunostaining for active β-catenin was performed in standard cell culture conditions (450 mg/dL) and under glucose deprivation for 48 h. Briefly, we harvested the cells (10,000 cells/200 µL), counted, washed thoroughly with PBS, and centrifuged on microscope slides using a Cytospin. Samples were then dried, fixated in 4% PFA, incubated in Tween 20 and blocked using 5% BSA. Primary antibody was incubated over night at 4 °C (active β-catenin 1:1000 in 5% BSA/TBST (non-phospho/active β-catenin, Ser33/37/Thr41, rabbit mAb; Cell Signaling, Danvers, MA, USA)). Subsequently, the samples were washed thoroughly using TBST and incubated for 1 h at room temperature with a secondary antibody goat anti-rabbit IRDye800CW (1:10,000, LI-COR #926-32211)). The cells were stained using DAPI, pictures were taken with a fluorescence microscope (Axiovision Apotome. Two-confocal microscope (Zeiss, Jena, Germnay)) and assessed by the software ZEN. The assessed fluorescence signals (green) translated into the amount of stained active β-catenin.

### 2.8. Quantitative Real-Time PCR (RT qPCR)

RNA isolation was performed using the RNeasy Mini Kit (Qiagen, Hilden, Germany), and cDNA synthesis using the M-MLV reverse transcriptase (Promega, Madison, WI, USA), M-MLV buffer (Promega), random hexamer primers, and Ribolock for RT-qPCR. SYBR Green Supermix (BioRad, Hercules, CA, USA), 10 ng cDNA, and 10 pmol primers were combined and run in a CFX Connect thermal cycler (BioRad). The expression of target genes was normalized to beta-2-microglobulin. Primer sequences used in this study were as follows: *β-catenin*: Fwd-GGGCCTCAGAGAGCTGAGTA, Rev-TGAGCAGCATCAAACTGTGTAG; *Axin2*: Fwd- AGCCAAAGCGATCTACAAAAGG, Rev-GGTAGGCATTTTCCTCCATCAC; *ZEB1*: Fwd-AAGAATTCACAGTGGAGAGAAGCCA, Rev-CGTTTCTTGCAGTTTGGGATT; *C-Myc*: Fwd-CCTTAATTAAAATGCCCCTCAACGTTAGCT, Rev- GGAATTCCATATGTTACGCACAAGAGTTCCGTA; and *MCT-1*: Fwd- GCTGGGCAGTGGTAATTGGA, Rev-CAGTAATTGATTTGGGAAATGCAT.

### 2.9. Whole Transcriptome Analysis

To obtain differential expression of genes, RNA was extracted and whole transcriptome analysis (3′ mRNA sequencing) was performed at the NGS Core Facility (Bonn, Germany). We applied the R package Deseq2 to identify differentially expressed genes in the control group versus the test/inhibitor group (dimethyl sulfoxide (DMSO) and/or LGK974) and used the R package clusterProfiler to recognize differentially expressed genes enriched in the KEGG pathways. Since DMSO alone has been shown to influence gene expression [21,22], we first attempted to exclude those genes which were primarily affected by the DMSO treatment. For this, we first filtered out the altered genes from the datasets that were only associated with DMSO treatment (standard glucose concentrations (Glc+) versus glucose withdrawal (Glc−). Subsequently, the obtained values were compared to the LGK974 treatment datasets (Glc+ versus Glc−).

### 2.10. LGK974 and DMSO Treatment

We assessed the viability of GSCs cultivated in glucose-depleted conditions and in the presence of the pharmacological WNT inhibitor LGK974 (a porcupine inhibitor (no. 1241454); Peprotech, Hamburg, Germany). LGK974 was dissolved in DMSO (Sigma-Aldrich) and used according to the manufacturer’s instructions. Specifically, we performed MTT assays on GSCs grown with and without glucose (450 and 0 mg/dL, respectively) and treated with five different LGK974 concentrations (GBM1 and JHH520: 20 µM, 10 µM, 5 µM, 2.5 µM, and 1.25 µM; BTSC233: 80 µM, 40 µM, 20 µM, 10 µM, and 5 µM). DMSO solvent controls (Glc+/Glc−) were used for normalization. We normalized the treatment groups to their corresponding controls after a 48 h incubation period.

### 2.11. In Vitro Drug Screening

An in vitro drug screen was conducted as previously reported in reference [23]. Using the Tecan D300e Digital Dispenser, a drug library, comprising 231 clinically approved chemotherapeutic drugs, was distributed in 384-well plates (Corning, Corning, NY, USA; Tecan, Männedorf, Switzerland). To determine the optimal number of cells to use in the screen, the growth curves of each cell line were analyzed before seeding them into the drug-coated plates. Cell viability was assessed by a GUAVA MUSE cytometer (Count and Viability, Luminex, Austin, TX, USA). Subsequently, an aliquot of 30 µL of cell suspension was dispensed into the prepared 384-well plates and incubated for 72 h. The CellTiter-Glo luminescent cell viability assay (Promega, Germany) was used to determine cell viability according to the manufacturer’s protocol and readout.

### 2.12. Gas Chromatography Mass Spectrometry: Cell Harvesting and Metabolite Extraction

Cells were cultivated under standard glucose concentration (450 mg/dL) (37 °C, 5% CO_2_) and washed twice with PBS before glucose-free Neurobasal medium was added to induce starvation. Starved cells were treated with LGK974 or DMSO for 48 h before being harvested by centrifugation (4 °C, 5 min, and 1000 rcf), counted, and washed with ice-cold 0.9% (*w*/*v*) sodium chloride (NaCl) solution. For cell disruption and metabolite extraction, 350 µL of a methanol: chloroform (10:4.28) solution was added. The samples were vortexed and incubated for 1 h at −20 °C. Subsequently, 560 µL of water containing the internal standard (5 µM ribitol) was added and the samples were vortexed and incubated on ice for 10 min. Samples were then centrifugated at maximum speed (12,000 rpm) for 2 min and two phases were obtained (an aqueous upper phase and a hydrophobic lower phase). The upper aqueous phase was dried by lyophilization. After resuspension in 250 µL methanol (50%), an aliquot of 50 µL was dried in a glass inlet for analysis by gas chromatography.

### 2.13. Gas Chromatography Mass Spectrometry

The samples were prepared and analyzed by gas chromatography mass spectrometry (GC-MS) analysis as previously reported in references [24,25]. Metabolites were identified by comparing obtained spectra to spectra in the NIST14 Mass Spectral Library (https://www.nist.gov/srd/nist-standard-reference-database-1a-v14) (retrieved on 10 May 2021) using the MassHunter Qualitative program (v.b08.00; Agilent Technologies, Santa Clara, CA, USA). In addition, a quality control sample including all target substances was analyzed. The MassHunter Quantitative program was used to combine the peaks (v.b08.00; Agilent Technologies). For relative quantitation, all metabolite peak areas were normalized to the peak area of the internal standard ribitol. A defined dose of LGK974 was used in all cell lines (GBM1: 10 µM, JHH520: 20 µM, BTSC233: 40 µM) in these experiments.

### 2.14. Statistical Analyses

All statistical tests were performed using unpaired Student’s *t*-tests using GraphPadPrism software, version 8.0 (GraphPad Software, San Diego, CA, USA). All results are presented as mean + SD of a minimum of three independent biological replicates. For all experiments, significance was defined as a *p* value below 0.05.

## 3. Results

### 3.1. Glucose Starvation Impacts Cell Viability and Invasive Potential of GSCs

**Figure 1:**
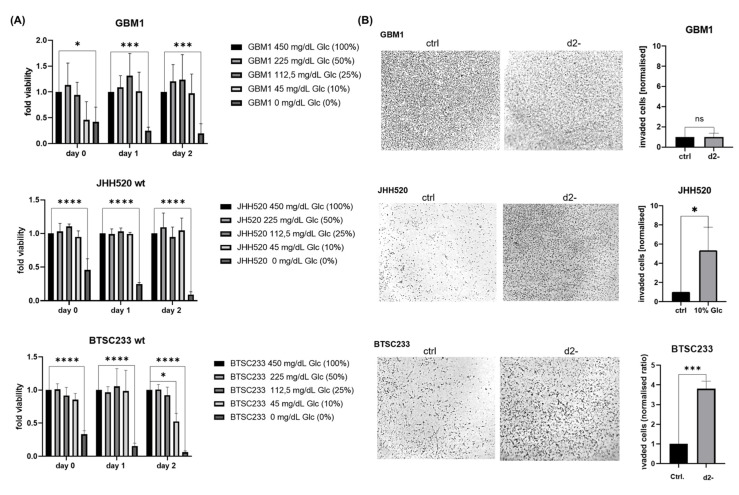
Glucose starvation decreases cell viability and enhances invasion in glioblastoma cell lines. (**A**) Glioblastoma cell lines (GBM1, JHH520, and BTSC233) were cultivated in standard cell culture glucose concentrations (450 mg/dL), decreased glucose concentrations (225 mg/dL, 112, 5 mg/dL, and 45 mg/dL) and in a glucose-depleted cell culture medium. The glucose concentration reduced to 45 mg/dL significantly decreased cell viability (*p* < 0.05) in BTSC233, whereas glucose depletion significantly inhibited viability in all cell lines (*p* < 0.0001). (**B**) A Boyden chamber assay was performed with all cell lines (GBM1, JHH520, and BTSC233) cultivated in standard (450 mg/dL) as a control condition (ctrl) and were compared to the corresponding starved samples (0 mg/dL) for 48 h (d2-). Glucose deprivation significantly enhanced invasion in JHH520 (*p* < 0.05) and BTSC233 cells (*p* < 0.001). The data are represented as mean + SD (*n* = 3). Statistical significance was calculated using an unpaired Student’s *t*-test. * *p* < 0.05, *** *p <* 0.001, and **** *p* < 0.0001.

First, we cultured GSCs in a Neurobasal medium containing glucose at varying concentrations (450 mg/dL, 225 mg/dL, 112.5 mg/dL, and 45 mg/dL) for 24 and 48 h (Figure 1A). In GBM1 and JHH520 cells, reduction in glucose levels (450 mg/dL–45 mg/dL) did not significantly affect cell viability. However, reducing the glucose concentration to 45 mg/dL significantly decreased the viability of BTSC233 cells (*p* < 0.05). When compared to the control (non-starved cells cultured at a standard glucose concentration of 450 mg/dL), reduced glucose levels showed little effect; however, complete glucose deprivation significantly reduced GSC’s viability. In addition, glucose-depleted cultures of JHH520 and BTSC233 cells displayed significantly enhanced invasion after 48 h compared to the control (JHH520 *p* < 0.05, BTSC233 *p* < 0.001) (Figure 1B). Interestingly, all cell lines showed a significant increase in clonogenic capacity when cultivated in reduced glucose concentration media (45 mg/dL) as opposed to standard cell culture conditions (450 mg/dL glucose) (GBM1 *p* < 0.001, JHH520 *p* < 0.01, and BTSC233 *p* < 0.05) (Supplementary Figure S1A).

### 3.2. Glucose Starvation Enhances WNT Activity and Induces Alterations in β-Catenin and Associated Genes

**Figure 2:**
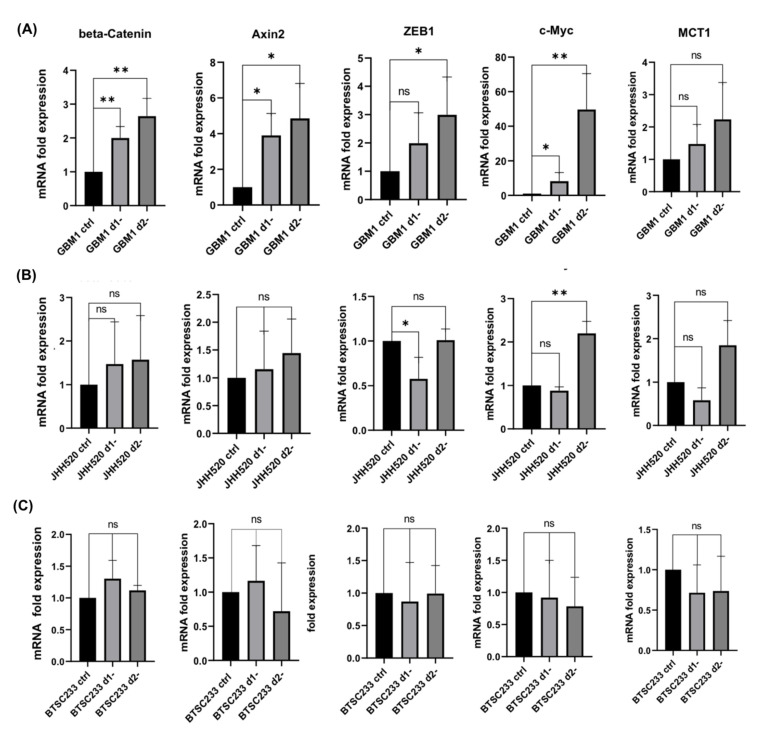
Differential mRNA expression of *CTNNB1* and β-catenin target genes in glucose-starved GSCs. (**A**–**C**) Differential mRNA expression of β-catenin and downstream genes of WNT signaling were assessed in three GSCs: GBM1, JHH520, and BTSC233 after 24 h (d1-) and 48 h (d2-) of glucose starvation. The data are presented as mean + SD (*n* = 3). Statistical significance was calculated using an unpaired Student’s *t*-test. * *p* < 0.05, ** *p* < 0.01.

WNT signaling plays an important role in mediating metabolic resistance in neoplastic tissues [14]. Therefore, we assessed the activity of the canonical WNT pathway in GSCs using the *TCF* luciferase reporter assay. A significant increase in luciferase signal intensity was observed in *TCF* luciferase-transfected GBM1 and JHH520 cells after 48 h of glucose withdrawal compared to controls (non-starved *TCF* luciferase-transfected cells) (*p* < 0.05). No change was observed in BTSC233 cells (Supplementary Figure S1B). The findings in GBM1 and JHH520 cells suggested that glucose deprivation in GSCs may be associated with the activation of the canonical WNT pathway. Therefore, we investigated the direct effect of glucose deprivation (24, 48 h) on β-catenin protein levels in wildtype GSCs. We observed significantly increased levels of β-catenin in JHH520 cells after 24 h of starvation (*p* < 0.05) (Supplementary Figure S1C). Both starved GBM1 and BTSC233 cells showed no significant change in β-catenin protein levels compared to the control group. Since transcription-related changes can be more sensitive compared to stable/conserved protein levels, we extended our analysis by evaluating the mRNA expression of β-catenin and WNT/β-catenin downstream genes (*AXIN2*, *ZEB1*, *MYC*, and *MCT1*) after 24 and 48 h of glucose deprivation (Figure 2). In GBM1 cells, we detected significantly upregulated *CTNNB1* (β-catenin), *AXIN2*, *ZEB1*, and *MYC* mRNA expression levels after starvation for 48 h (*p* < 0.01). A 24 h period of starvation in GBM1 cells also induced a significant upregulation of *CTNNB1*, *AXIN2*, and *MYC* mRNA expression, but not of *ZEB1* transcripts. It was also found that *ZEB1* was significantly downregulated (24 h of treatment), while *MYC* was upregulated at the mRNA level in JHH520 cells (48 h). BTSC233 cells did not show alterations in the mRNA expression of these selected genes. The transcript levels of *MCT1* were not altered in any of these cell lines.

### 3.3. Pharmacological WNT Inhibition Sensitizes GSCs to Glucose Starvation-Induced Cell Death and Moderately Affects Gene Expression of β-Catenin and Associated Genes

**Figure 3:**
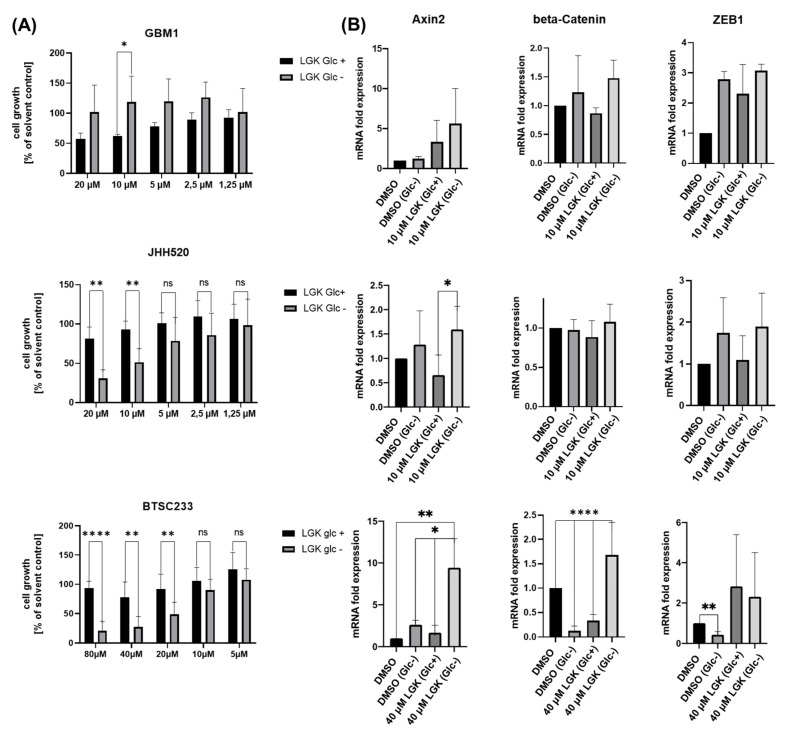
LGK974 sensitizes GSCs to glucose starvation-induced cell death and affects the mRNA expression of *CTNNB1* and associated genes *AXIN-2* and *ZEB1*. (**A**) We assessed the viability of our GSC lines cultured in standard glucose concentrations of 450 mg/dL (Glc+) or in a glucose-depleted standard cell culture medium of 0 mg/dL (Glc−) and simultaneously performed the pharmacological inhibition of WNT signaling with LGK974 (LGK) for a time period of 48 h. DMSO solvent controls were used for normalization. In order to normalize to the solvent control, equivalent amounts of DMSO were used in all conditions per cell line. LGK974 significantly decreased the viability of glucose-deprived JHH520 cells at 10 µM and 20 µM (*p* < 0.01), and at 20 µM, 40 µM (*p* < 0.01), and 80 µM (*p* < 0.0001) in BTSC233 compared to cells that have been treated with LGK974 but which have not been depleted of glucose (LGK Glc+). GBM1 glucose-starved cells that were simultaneously treated with LGK974 displayed increased viability (*p* < 0.01) when treated with 10 µM LGK974 compared to cells that have been treated with LGK974 but which have not been depleted of glucose (LGK Glc+). (**B**) We also assessed the mRNA expression of *AXIN2*, *CTNNB1* (β-Catenin), and *ZEB1* of our GSC lines GBM1, JHH520, and BTSC233 after a time period of 48 h of treatment with DMSO (equivalent in all conditions per cell line) and in a standard glucose concentration of 450 mg/dL as a control (DMSO). Additionally, cells have been depleted of glucose and cultivated in DMSO (DMSO Glc−), treated with LGK974 (LGK) in defined (assessed concentrations inducing significant change of viability when depleted of glucose per cell line: GBM1: 10 µM LGK974; JHH520: 10 µM LGK974; and BTSC233: 40 µM LGK974) standard glucose concentrations (LGK Glc+) and under glucose withdrawal (LGK Glc−). *AXIN2* mRNA levels were significantly increased in JHH520 and BTSC233 cells treated with LGK974 and depleted of glucose (*p* < 0.05–*p* < 0.01). The data are presented as mean + SD (*n* = 3). Statistical significance was calculated using an unpaired Student’s *t*-test. * *p* < 0.05, ** *p* < 0.01, and **** *p* < 0.0001.

Next, we assessed the viability of cell lines cultivated in glucose-depleted conditions and under pharmacological WNT inhibition with LGK974 (Figure 3A). In JHH520 cells, treatment with LGK974 (10 µM and 20 µM) significantly decreased the viability of glucose-depleted cells (*p* < 0.01). In BTSC233 cells, high concentrations of LGK974 (80 µM, 40 µM, and 20 µM) and glucose starvation significantly reduced cell viability (*p* < 0.01–*p* < 0.0001), suggesting that WNT signaling mediates metabolic resilience to maintain viability under nutrient/metabolic stress. Transcriptional changes in WNT/β-catenin target genes (*AXIN2, ZEB1*) were observed in JHH520 and BTSC233 cells (*p* < 0.05) upon treatment with LGK974 or control (DMSO) and simultaneous glucose deprivation (Figure 3B).

**Figure 4:**
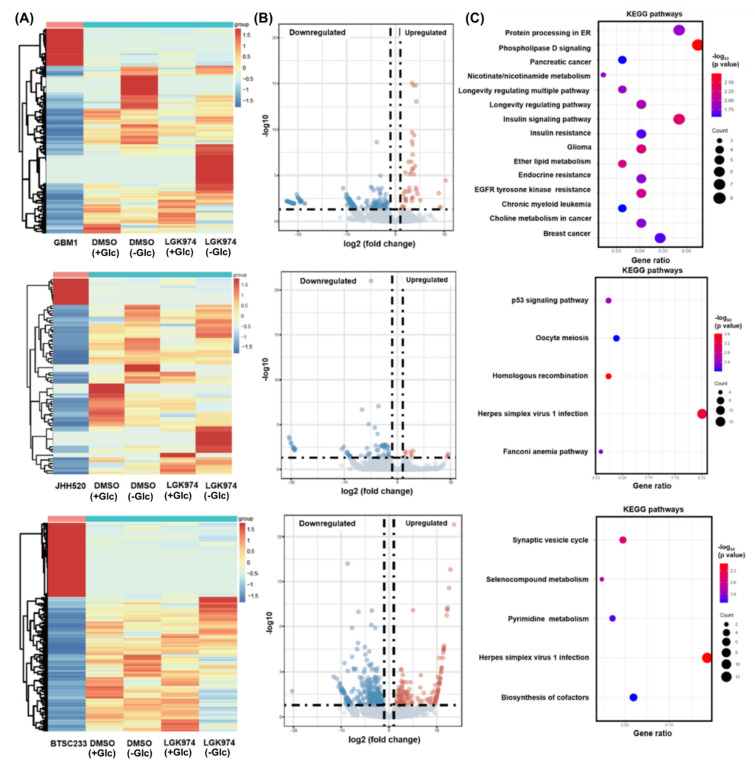
Transcriptome-wide gene expression analysis of GBM1, JHH520, and BTSC233 cells. (**A**) A heatmap showing variations in gene expression in cells treated with DMSO as solvent control and LGK974 in standard cell culture glucose concentration (450 mg/dL) and under complete glucose deprivation. (**B**) Altered genes in treated compared to untreated cell lines and (**C**) KEGG-associated pathways.

To obtain a comprehensive overview of the transcriptional changes beyond WNT/β-catenin target genes, we next performed a genome-wide transcriptional analysis. When comparing samples treated with DMSO (Glc+/−) and/or LGK974 (Glc+/−), both upregulated and downregulated gene clusters were found in all three cell lines (Figure 4A,B). Among them, significantly altered genes were: GBM1: up-regulated genes: *RYR1, HS6ST2*, and *C14orf132*, down-regulated genes: *TACSTD2, VAMP8*, and *BPIFA1*; JHH520: up-regulated genes: *PAWR*, *TIMP3*, and *HOXB7*, down-regulated genes: *ABCG2*, *TSACC*, and *PYY*; BTSC233: upregulated genes: *NCAN*, *SHC2*, and *NF1*, downregulated genes: *SDK1*, *TFPI2*, and *NBDY.* Furthermore, KEGG pathway analysis showed that these differentially expressed genes (DEGs) were highly associated with pathways ranging from metabolism to cancer (Figure 4C). Of interest, we also identified DEGs within the WNT/β-catenin pathway that were induced by glucose deprivation (Supplementary Figure S2). In addition, we evaluated the expression of β-catenin-dependent and independent target genes, retrieved from a recent study [26], and found them altered in our datasets (Supplementary Figure S3).

We next performed immunostaining for active β-catenin in GSCs (GBM1, BTSC233) under glucose deprivation for 48 h (Supplementary Figure S4). Staining increased in cells deprived of glucose, indicating a quantitatively higher amount of transcriptionally active β-catenin. Likewise, *TCF* luciferase activity was increased significantly in GBM1 and JHH520 cells after 48 h of glucose deprivation, whereas a tendency toward the upregulation of *TCF* luciferase activity was observed in BTSC233 cells.

### 3.4. Treatment with LGK974 and/or Glucose Starvation Alters Intracellular Metabolite Concentrations

**Figure 5:**
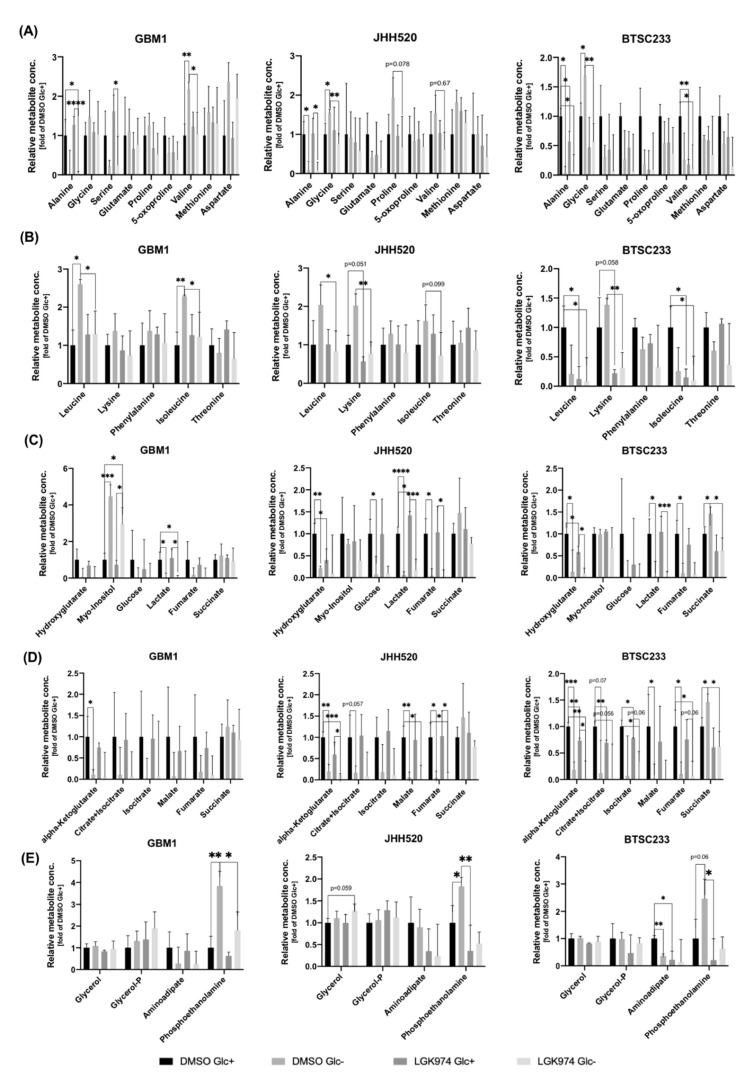
LGK974 treatment and/or glucose starvation-altered intracellular metabolite concentrations as determined by GC-MS. The effect on intracellular metabolite concentrations in cells treated with DMSO and LGK974 in standard cell culture glucose concentration (450 mg/dL) (DMSO Glc+ (as a solvent control) and LGK974 Glc+) and under complete glucose deprivation for a time period of 48 h (DMSO Glc− and LGK974 Glc−) is shown. Quantitative characterization of diverse intracellular metabolites such as (**A**) glucogenic amino acids (alanine, glycine, serine, glutamate, (5-oxo-)proline, valine, methionine, and aspartate), (**B**) ketogenic amino acids (leucine, lysine, phenylalanine, isoleucine, and threonine), (**C**) oncometabolites (hydroxyglutarate, myo-inositol, glucose, lactate, fumarate, and succinate), (**D**) tricarboxylic acid (TCA) metabolites (alpha-ketoglutarate, citrate, isocitrate, malate, fumarate, and succinate) and (**E**) lipophilic metabolites (glycerol(-P), aminoadipate, and phosphoethanolamine) were evaluated. The data are presented as mean + SD (*n* = 3). The y-axis depicts the relative metabolite concentration in the intracellular compartment (normalized to the corresponding solvent control (DMSO Glc+)). Statistical significance was calculated using an unpaired Student’s *t*-test. * *p* < 0.05, ** *p* < 0.01, *** *p* < 0.001, and **** *p* < 0.0001.

Next, we performed gas chromatography mass spectrometry (GC-MS) to analyze intracellular metabolites in GSC lines treated with LGK974 or DMSO for a time period of 48 h under standard (450 mg/dL) and depleted glucose concentrations (0 mg/dL) (Figure 5). The intracellular level of the glucogenic amino acid alanine was significantly (*p* < 0.0001–*p* < 0.05) reduced under glucose deprivation in our GSC lines irrespective of LGK974 treatment (Figure 5A). We also observed a reduction of valine (GBM1 *p* < 0.05, JHH520 *p* = 0.67, and BTSC233 *p* < 0.05). Other glucogenic amino acids such as glutamate, 5-oxoproline, methionine, and aspartate showed no changes. Treatment with LGK974 combined with glucose deprivation impacted ketogenic amino acids (leucine, lysine, phenylalanine, isoleucine, and threonine) (Figure 5B). Leucine (in BTSC233 *p* < 0.05) and lysine (JHH520: *p* = 0.051, BTSC233: *p* = 0.058) were found to be reduced upon LGK974 treatment alone. Isoleucine was reduced upon LGK974 treatment in BTSC233 cells (*p* < 0.05), while simultaneous WNT inhibition and glucose deprivation resulted in the reduction of isoleucine in all cell lines (GBM1 and BTSC233: *p* < 0.05; JHH520: *p* = 0.099). Both phenylalanine and threonine were not changed significantly.

WNT inhibition by LGK974 also reduced the intracellular concentration of the oncometabolite hydroxyglutarate, particularly in JHH520 cells (*p* < 0.0001) and BTSC233 cells (*p* < 0.05) (Figure 5C). Succinate levels were increased by DMSO and glucose deprivation in BTSC233 cells; however, the combined use of LGK974 and glucose starvation resulted in the suppression of succinate levels. Starvation resulted in decreased intracellular levels of glucose, lactate, and fumarate in JHH520 cells (*p* < 0.0001) and BTSC233 cells (*p* < 0.05), irrespectively of LGK974 treatment. Myoinositol, a well-known oncometabolite in glioblastoma, was significantly elevated in starved GBM1 cells (*p* < 0.001). Glucose starvation also led to significantly reduced levels of tricarboxylic acid (TCA) metabolites, such as alpha-ketoglutarate, in GSCs treated with DMSO (GBM1: *p* < 0.01, JHH520: *p* < 0.01, and BTSC233: *p* < 0.05) and LGK974 (JHH520: *p* < 0.05, BTSC233: *p* < 0.05) (Figure 5D). Some TCA metabolites (citrate, isocitrate, malate, and fumarate) were significantly reduced after the glucose starvation of JHH520 and BTSC233 cells (*p* < 0.001–*p* = 0.057). Glucose deprivation led to an increased intracellular level of phosphoethanolamine in all cell lines (*p* < 0.0001–*p* < 0.001), irrespectively of WNT inhibition (Figure 5E).

### 3.5. In Vitro Drug Screen in Glucose-Deprived GSCs

To determine the effects of nutritional stress on chemosensitivity, we performed an in vitro drug screen comprising 231 proven chemotherapeutic drugs using two GSCs enriched cell lines (GBM1, BTSC233) grown in standard cell culture conditions and under glucose deprivation (Supplementary Figure S5). Staurosporin, a global kinases inhibitor was used as a positive control. The analysis showed that eleven drugs sensitized the GSCs to starvation-induced cell death—including LGK974 and berberine, which both impair the WNT pathway. In addition to these, disulfiram (aldehyde dehydrogenase inhibitor), andrographolide (NFkB inhibitor), auranofin bacterial (inhibitor of bacterial thioredoxin reductase), pazopanib (multitargeted tyrosine kinase inhibitor), entinostat (histone deacetylase inhibitor), honokiol (ERK inhibitor), ravoxertinib (ERK inhibitor), rigosertib sodium (PI3K- and Polo-like Kinase inhibitor), and masitinib mesylate (c-Kit, FGFR, PDGFR, Scr inhibitor) were also identified as drugs that sensitize to starvation-induced cell death.

## 4. Discussion

Although aberrant WNT activation has been associated with malignant transformation in various cancers, its role in glioblastoma is yet to be fully unveiled. WNT signaling mediates the clonogenicity and growth of neural progenitor cells and mediates chemoresistance to alkylating agents such as Temozolomide (TMZ) in glioblastoma [27]. Due to its role in promoting metabolic plasticity, WNT upregulation has been postulated as a mediator of metabolic resilience in malignant tissues [28]. Thus, our study focused on the WNT-mediated response to glucose deprivation in GSCs. Initially, we assessed the viability of glucose-starved GSCs by performing an MTT assay, thereby determining the percentage of cells surviving extreme metabolic stress. A small subpopulation of cells managed to survive in a glucose-deprived microenvironment (Figure 1). In our next experiment, we assessed invasive capacities after glucose starvation and showed that the depletion of glucose led to the acquisition of an invasive phenotype, possibly by inducing a mesenchymal transition (Figure 3). This acquisition of an invasive phenotype in nutrient-limiting microenvironments has been shown across various cancer tissues before [29].

Furthermore, we performed a soft agar colony formation assay and quantified the self-renewal properties as a surrogate of the stem-like cell phenotype, observing the increased clonogenic potential of GSCs that were exposed to low glucose concentrations for four weeks. This indicates an enrichment of GSCs by nutrient deprivation. A similar advantage in survival leading to the selection of stem-like cancer cells has already been observed after chemo- and radiotherapy [30]. The impact of glucose starvation in our analysis was also evident on both the local (*β-catenin* and target genes) and the global (whole-genome transcriptome) genomic level. Moreover, the significant increase in luciferase signal also suggested an increased activity of the canonical WNT pathway following glucose starvation. Based on our observations, we concluded that glucose-depleted GSCs activate canonical WNT signaling in response to metabolic stress. Indeed, simultaneous WNT inhibition with the porcupine inhibitor LGK974, which interferes with the endoplasmatic release of WNT molecules, led to significantly decreased viability under glucose starvation in a dose-dependent manner. Although this phenomenon can predominantly be attributed to direct WNT/β-catenin inhibition, off-target effects of LGK974 cannot be excluded. Furthermore, heterogenic transcriptional characteristics of GSCs used in this study (GBM1: adult male, classical, TP53 p.L130I, IDH-wildtype; JHH520: adult female, mesenchymal, TP53 p.H179D, IDH-wildtype; and BTSC233: adult female, mesenchymal, IDH-wildtype) may contribute to minor differences in observed responses towards metabolic stress. Such inherited heterogeneity between cancer cell lines leading to experimental discrepancies has previously been discussed [31].

In vitro drug screening identified LGK974 and berberine (WNT inhibitor used for hypercholesterinemia, diabetes, and hypertension [32]) as drugs that sensitize cells to starvation-induced cell death. In addition to that, we characterized the metabolic profile under glucose starvation, WNT inhibition, and a combination of WNT inhibition and glucose starvation, by utilizing GC-MS. We observed significant changes in various metabolites under WNT inhibition alone and in combination with glucose depletion. The glucogenic amino acid alanine was significantly reduced in starved cell lines, most likely due to anaplerosis [33], whereas other glucogenic amino acids (such as glutamate, 5-oxoproline, methionine, and aspartate) showed no changes. Whether these effects are confined to the metabolic microenvironment of gliomas or common to other cancers requires further studies. Notably, in all cell lines, ketogenic amino acids lysine and leucine were reduced, even more significantly under simultaneous WNT inhibition and glucose starvation. This indicates an additional role of WNT signaling in ketogenesis by fatty acid oxidation, which has also previously been reported [34]. Our results contribute to a more comprehensive understanding of WNT signaling as an important player in the regulation of ketogenesis not only through the beta-oxidation of fatty acids but also by the utilization of ketogenic amino acids. The impaired survival of glucose-deprived GSCs by simultaneous WNT inhibition may be due to the limited availability of ketogenic amino acids for the consequent utilization of anaplerotic reactions in the tricarboxylic acid cycle. In particular, alpha-ketoglutarate, citrate and isocitrate, malate, and fumarate were decreased in starved GSCs independent of WNT inhibition, suggesting a general mechanism, such as the rapid incorporation into the reaction chains of the tricarboxylic acid cycle. In addition, WNT inhibition of glucose-deprived GSCs affected several oncometabolites, such as hydroxyglutarate, myo-inositol, succinate, and fumarate. Hydroxyglutarate is an oncometabolite that accumulates in IDH-mutant glioma cells and correlates with poor prognosis [35]. In our study, hydroxyglutarate was significantly reduced under LGK974 treatment, suggesting a role of the WNT pathway in regulating oncometabolic-driven cancer growth. WNT inhibition decreased the phosphoethanolamine concentration, which was elevated by glucose starvation. A recent study showed that mutant IDH1 gliomas downregulate the synthesis of phosphocholine and phosphoethanolamine in a 2-hydroxyglutarate-dependent manner [36]. Although WNT activation played an important role in maintaining cell survival under extreme nutrient restriction, the exact mechanism by which glucose deprivation can enhance β-catenin activity remains unclear. Previously, it has been discussed that the degradation of β-catenin upon glucose deprivation is GSK3β-independent and mainly involves the protein kinase C (PKC)-dependent pathway [37]. Likewise, AMP-activated protein kinase (AMPK) phosphorylates β-catenin at Ser 552 [38] and further regulates its transcriptional level via phosphorylated histone deacetylase 5 (HDAC5) [39].

In summary, our findings reveal novel pleiotropic effects of WNT signaling on metabolic activity in glioblastoma stem-like cell lines. Metabolic fingerprints might possibly complement classic biomarkers, allowing for the better prediction of tumor behavior and clinical prognosis. Considering that in vitro tumor models do not closely mimic the in vivo tumor microenvironment, further analysis of patient-derived samples, especially from perinecrotic, oxygen- and nutrient-deprived tumor compartments is of paramount importance for the development of more efficient therapeutic strategies.

## 5. Conclusions

Our findings suggest that WNT activation plays an important role in promoting the survival of glioblastoma cells under extreme nutrient restrictions. Whether this is limited to malignant glioma-derived cell lines or is a general mechanism in cancer biology requires further attention.

## Data Availability

The data presented in this study are available in this article (and Supplementary Material).

## Supplementary Materials

The following supporting information can be downloaded at: https://www.mdpi.com/article/10.3390/cancers14133165/s1, Figure S1: (A) glucose starvation enhances the clonogenic capacity in the starved population of cells, (B) *TCF* luciferase activity, and (C) protein expression of active beta-catenin (ABC) in three cell lines; Figure S2: a heat map shows differentially expressed genes (DEGs) within the WNT/β-catenin pathway induced by glucose deprivation; Figure S3: a heat map shows the expression pattern of β-catenin-dependent and independent target genes (retrieved from reference [25]) in our datasets; Figure S4: immunostaining for active β-catenin in glioma cell lines in standard cell culture conditions and under glucose deprivation, (A) immunostaining for active β-catenin in GBM1 and BTSC233 in standard cell culture conditions (450 mg/dL) and under glucose deprivation for 48 h, (B) glucose deprivation enhances *TCF* luciferase activity (WNT activity); Figure S5: in silico drug screen in GBM cell lines with (A) staurosporin, (B) berberine, and (C) LGK974. (D) Other potential chemotherapeutics assessed via robot technology are also shown. Original western blot images from Figure S1C are included.

## Abbreviations

Axin2: axis inhibition protein 2; CNS: central nervous system; CSC: cancer stem-like cells; DMSO: dimethyl sulfoxide; EGF: epidermal growth factor; FGF: fibroblast growth factor; Fwd: forward; GC-MS: gas chromatography mass spectrometry; GLUT1: glucose transporter 1; IDH1: isocitrate dehydrogenase 1; LDH-A: lactate dehydrogenase A; MCT-1: monocarboxylate transporter 1; MTT: 3-(4,5-dimethylthiazol-2-yl)-2,5-diphenyltetrazolium bromide; NBT: nitro blue tetrazolium chloride; PDH: pyruvate dehydrogenase; PKM2: pyruvate kinase M2; Rev: reverse; rpm: rounds per minute; RT-qPCR: real-time quantitative PCR; TCA: tricarboxylic acid; *TCF*/*LEF*: T cell factor/lymphoid enhancer factor family; TMZ: temozolomide; WHO: World Health Organization; WNT: wiSuadngless and Int-1; ZEB1: zinc finger E-box binding homeobox 1.
