# Supplementary material for: WNT/β-Catenin-Mediated Resistance to Glucose Deprivation in Glioblastoma Stem-like Cells
Source: Cancers (Basel). 2022 Jun 28;14(13):3165. doi: 10.3390/cancers14133165 (PMC9264876; doi:10.3390/cancers14133165)
Supplement: Supplementary file 1 [file cancers-14-03165-s001.zip › cancers-1745878-non-published.pdf]

WB Data as requested by Rowang Wang

Manuscript ID: cancers-1498503

By Suad Yusuf

Supplementary

Figure S1C:

Representative blot

A

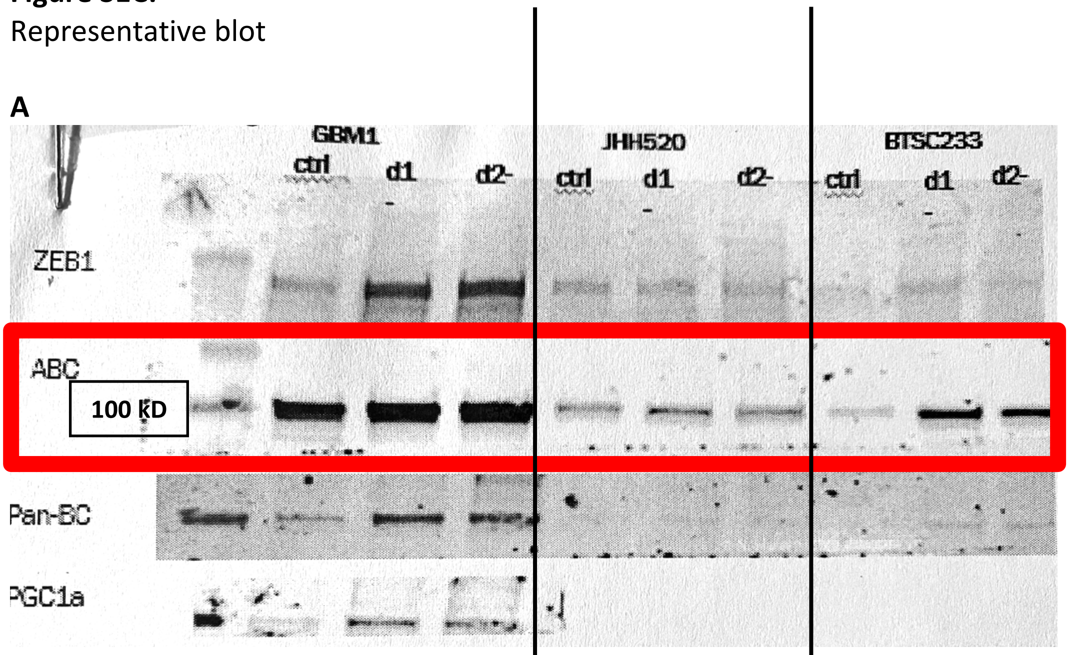

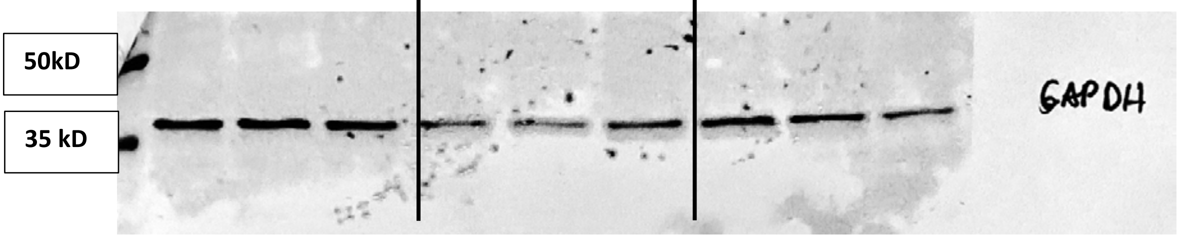

B

C

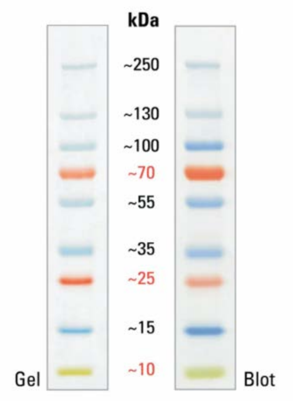

Antibodies:

Primary antibodies: active beta-catenin 1:1000 (Non-phospho/Active beta-catenin, Ser33/37/Thr41, rabbit mAb, Cell Signalling, Danvers, MA) (A) and GAPDH 1:5000 (GAPDH, D4C6R, mouse mAb, Cell Signalling, Danvers, MA) (B)were diluted in 5% BSA and incubated overnight at 4°C on the membranes. Secondary antibodies: goat anti-rabbit IRDye800CW (1:10000, LI-COR #926-32211) and goat anti-mouse IRDye680RD (1:10000, LI-COR #926-68070) were diluted in 5%BSA and incubated for 1 hour at room temperature.

WB ladder:

Thermo Scientific™ PageRuler™ Plus Prestained Protein Ladder, 10 bis 250 kDa (C)

Densitometry Data for the exemplary Blot shown were as following:
The fluorescence was assessed using LI-COR Odyssey CLx imager followed by Densi-tometry. GAPDH was used as a housekeeping protein and used for normalization.

D

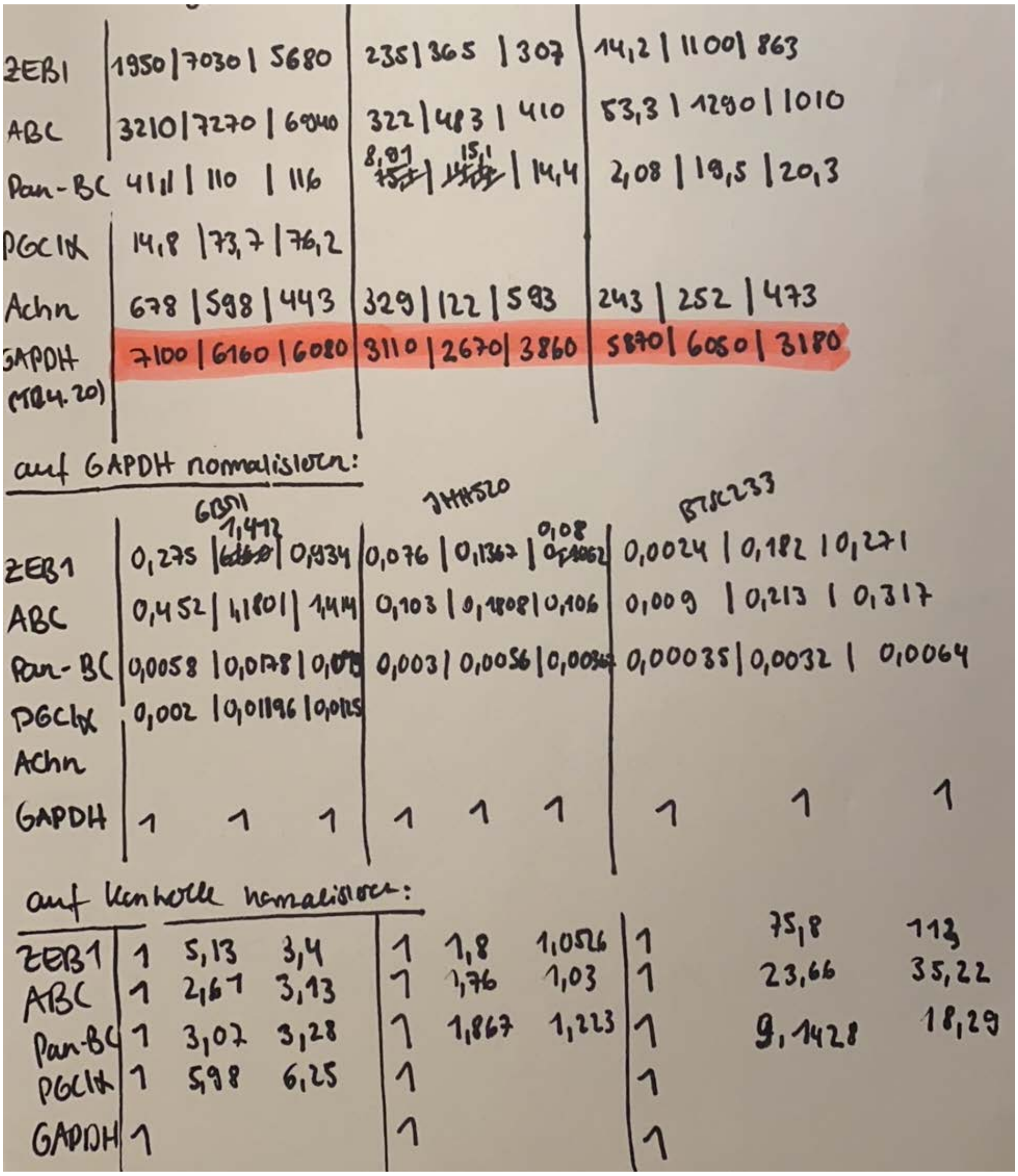

ZEB1	1950	7030	5680	235	365	307	14,2	1100	863
ABC	3210	7270	6940	322	483	410	53,3	1290	1010
Pan-BC	41,1	110	116	~~[illegible]~~ 8,81	~~[illegible]~~ 15,1	14,4	2,08	19,5	20,3
PGC1α	14,8	73,7	76,2						
Achn	678	598	443	329	122	593	243	252	473
GAPDH (M[illegible]4.20)	7100	6160	6080	3110	2670	3860	5870	6050	3180

auf GAPDH normalisieren:

	GBM1			JHH520			BTSC233		
ZEB1	0,275	~~[illegible]~~ 1,447	0,934	0,076	0,1367	~~[illegible]~~ 0,08	0,0024	0,182	0,271
ABC	0,452	1,1801	1,414	0,103	0,1808	0,106	0,009	0,213	0,317
Pan-BC	0,0058	0,0178	0,0[illegible]	0,003	0,0056	0,003[illegible]	0,00035	0,0032	0,0064
PGC1α	0,002	0,01196	0,0125						
Achn									
GAPDH	1	1	1	1	1	1	1	1	1

auf Kontrolle normalisieren:

ZEB1	1	5,13	3,4	1	1,8	1,0526	1	75,8	11[illegible]
ABC	1	2,61	3,13	1	1,76	1,03	1	23,66	35,22
Pan-BC	1	3,07	3,28	1	1,867	1,223	1	9,1428	18,29
PGC1α	1	5,98	6,25	1			1		
GAPDH	1			1			1		
