# Supplementary figures and images for: WNT/β-Catenin-Mediated Resistance to Glucose Deprivation in Glioblastoma Stem-like Cells
Source: Cancers (Basel). 2022 Jun 28;14(13):3165. doi: 10.3390/cancers14133165 (PMC9264876; doi:10.3390/cancers14133165)

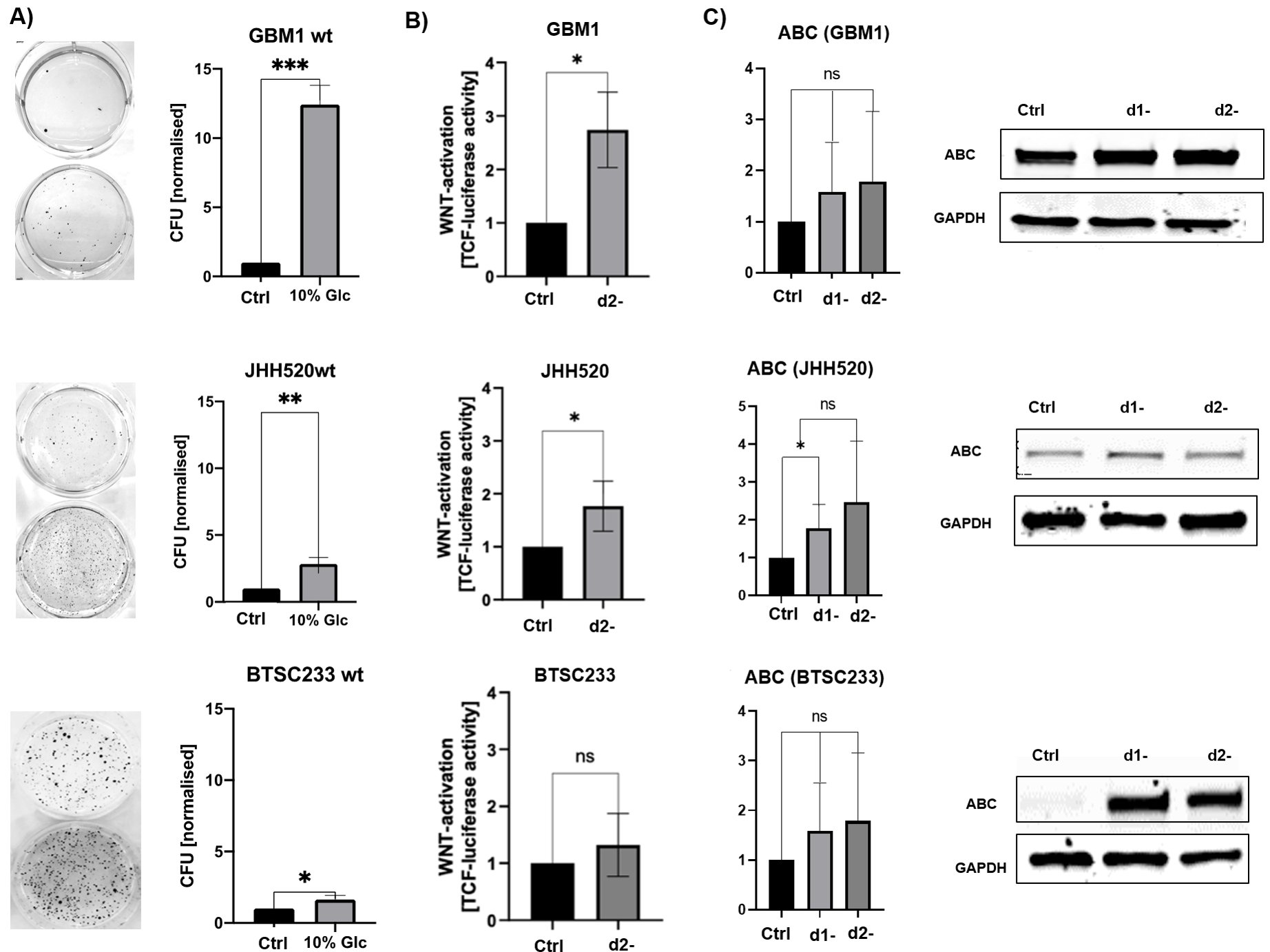

Supplement: Supplementary file 1 [file cancers-14-03165-s001.zip › Supplementary figure-S1.png]

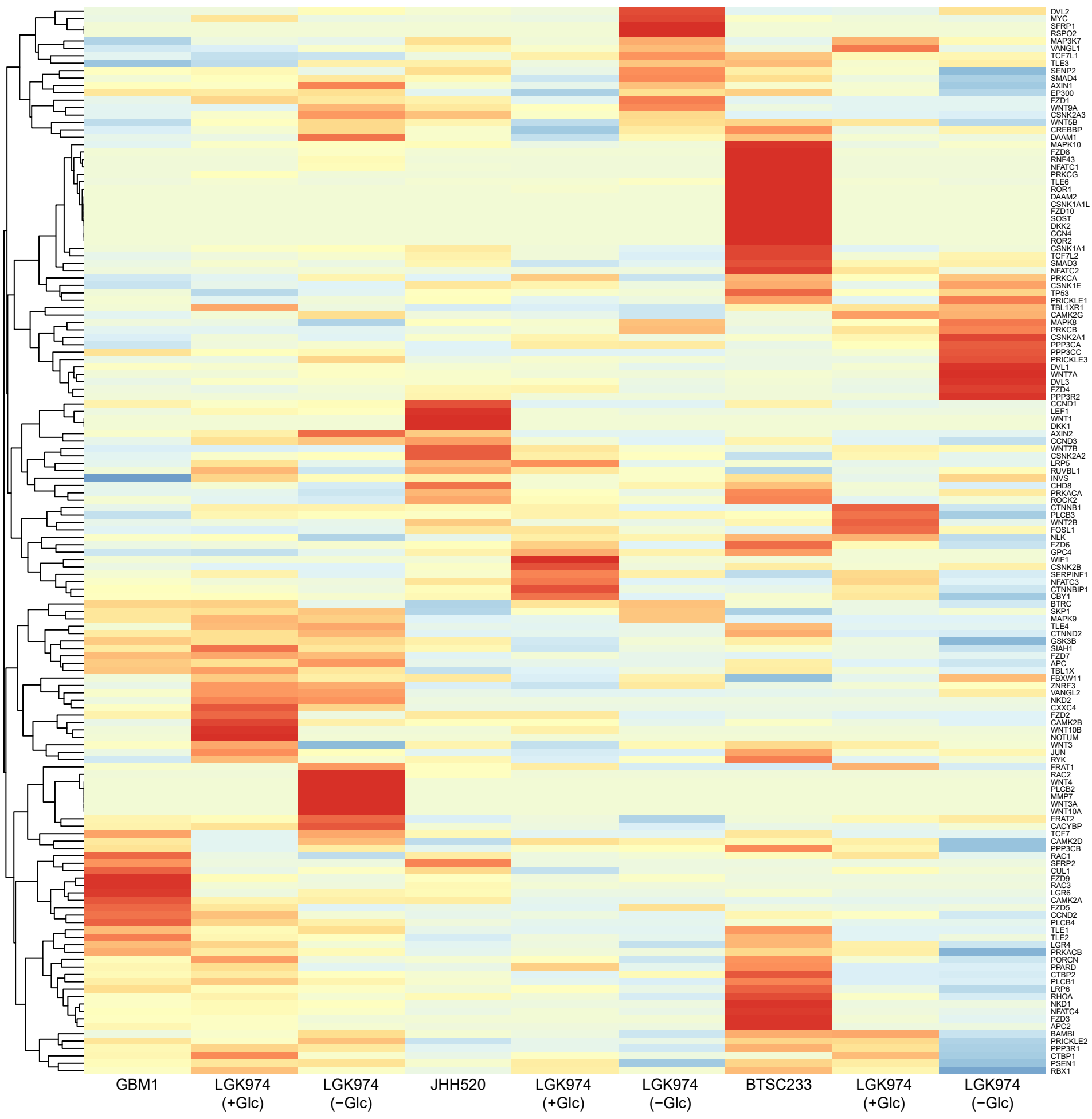

Supplement: Supplementary file 1 [file cancers-14-03165-s001.zip › Supplementary figure-S2.pdf]

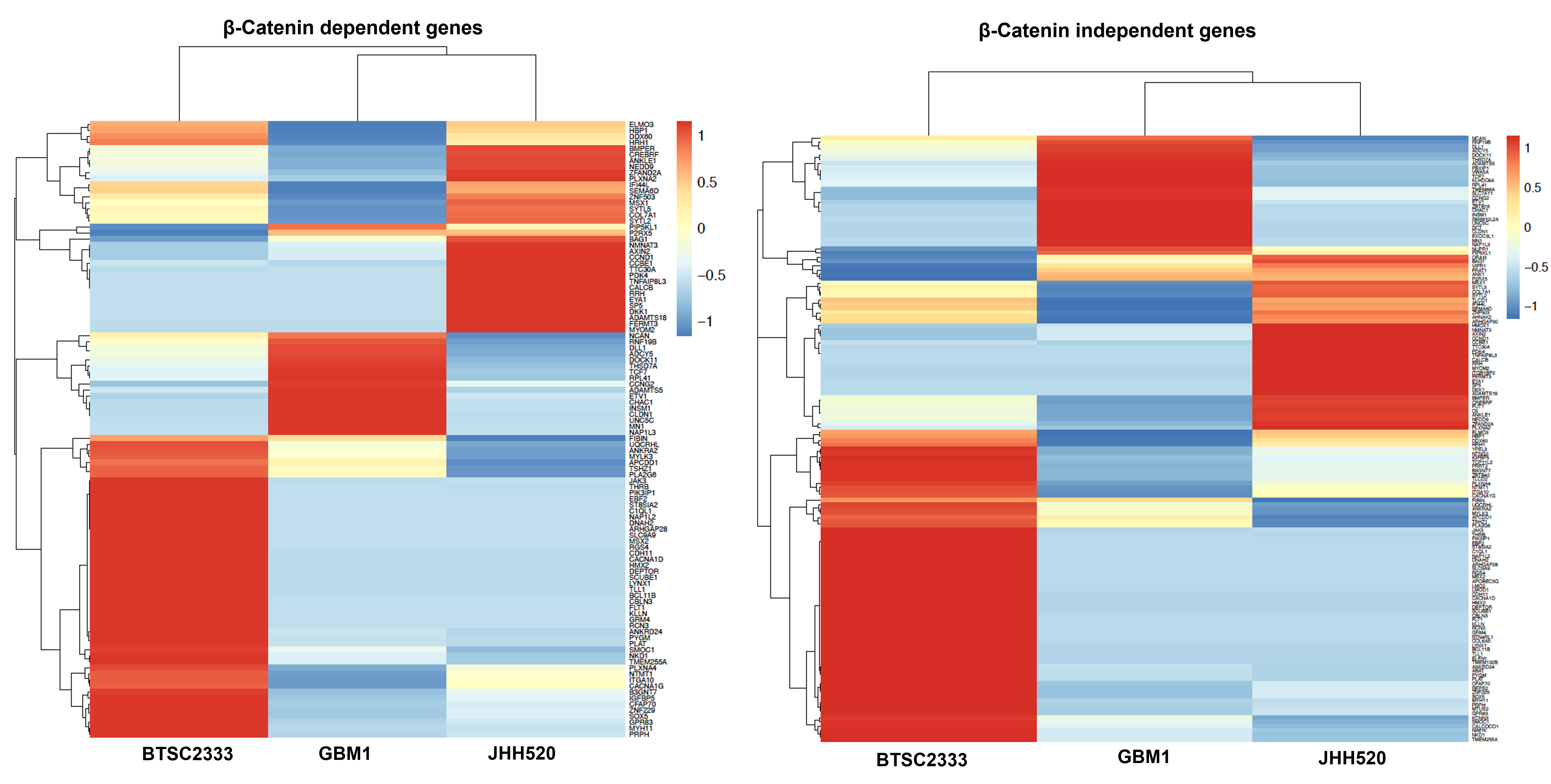

Supplement: Supplementary file 1 [file cancers-14-03165-s001.zip › Supplementary figure-S3.png]

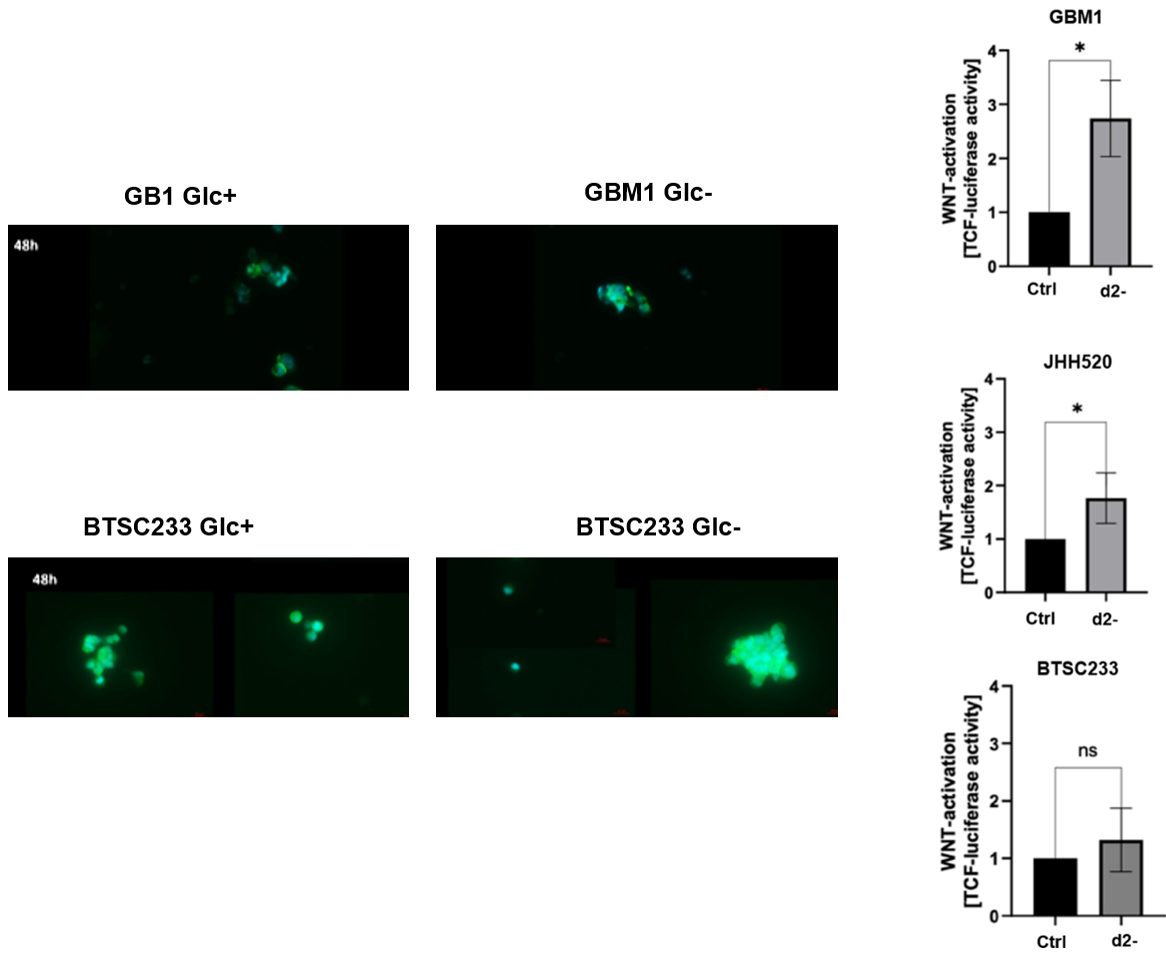

Supplement: Supplementary file 1 [file cancers-14-03165-s001.zip › Supplementary figure-S4.png]

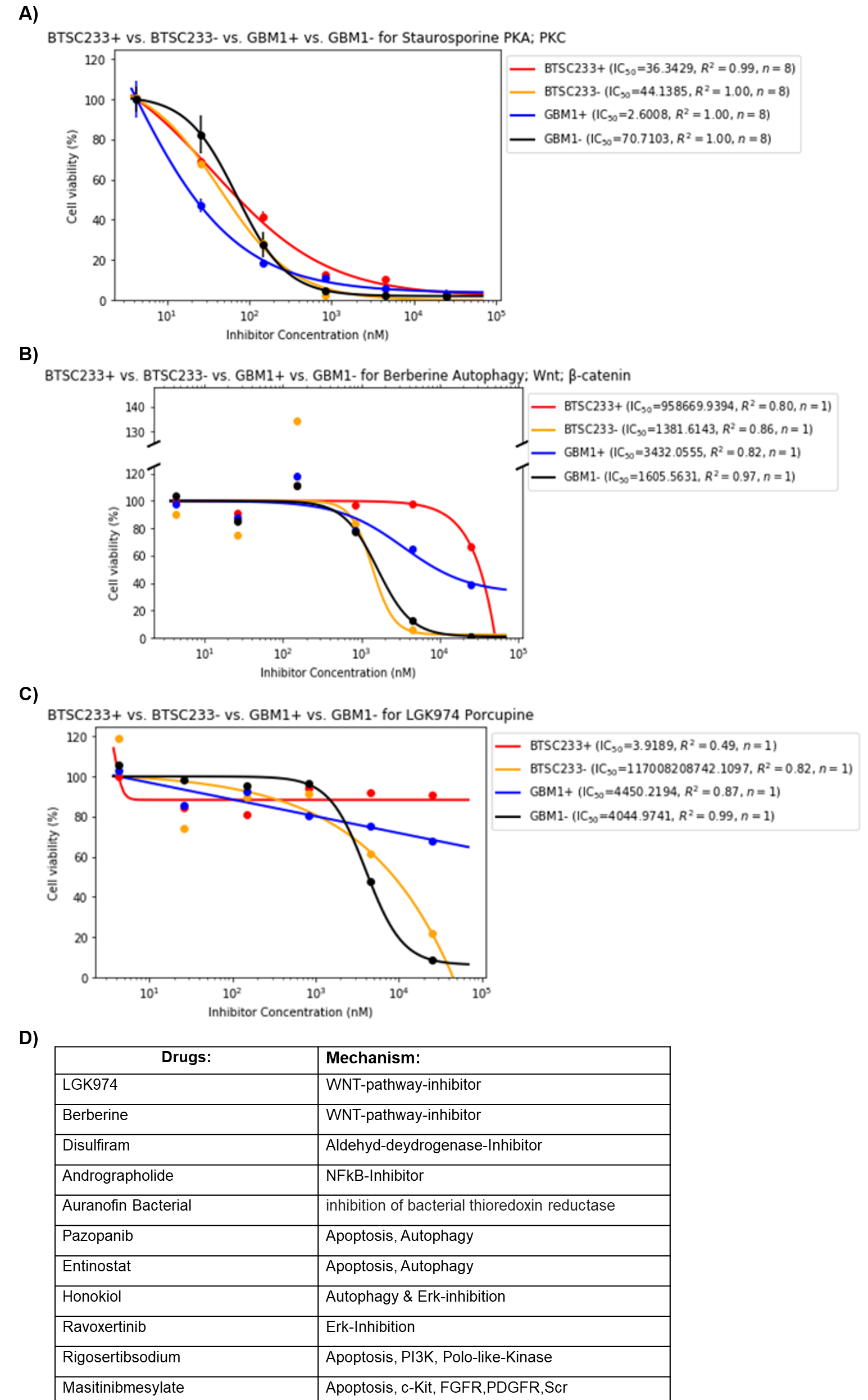

Supplement: Supplementary file 1 [file cancers-14-03165-s001.zip › Supplementary figure-S5.png]
